# A parallel spatiotemporal saliency and discriminative online learning method for visual target tracking in aerial videos

**DOI:** 10.1371/journal.pone.0192246

**Published:** 2018-02-13

**Authors:** Amirhossein Aghamohammadi, Mei Choo Ang, Elankovan A. Sundararajan, Ng Kok Weng, Marzieh Mogharrebi, Seyed Yashar Banihashem

**Affiliations:** 1 Institute of Visual Informatics, Universiti Kebangsaan Malaysia, Bangi, Selangor, Malaysia; 2 Center for Software Technology and Management (SOFTAM), Faculty of Information Science and Technology, University, Kebangsaan Malaysia, Bangi, Selangor, Malaysia; 3 Industrial Design Centre, Sirim Berhad, Selangor, Malaysia; 4 Department of Electrical and Computer Engineering, Buien Zahra Technical University, Buien Zahra, Iran; National Institute of Technology Rourkela, INDIA

## Abstract

Visual tracking in aerial videos is a challenging task in computer vision and remote sensing technologies due to appearance variation difficulties. Appearance variations are caused by camera and target motion, low resolution noisy images, scale changes, and pose variations. Various approaches have been proposed to deal with appearance variation difficulties in aerial videos, and amongst these methods, the spatiotemporal saliency detection approach reported promising results in the context of moving target detection. However, it is not accurate for moving target detection when visual tracking is performed under appearance variations. In this study, a visual tracking method is proposed based on spatiotemporal saliency and discriminative online learning methods to deal with appearance variations difficulties. Temporal saliency is used to represent moving target regions, and it was extracted based on the frame difference with Sauvola local adaptive thresholding algorithms. The spatial saliency is used to represent the target appearance details in candidate moving regions. SLIC superpixel segmentation, color, and moment features can be used to compute feature uniqueness and spatial compactness of saliency measurements to detect spatial saliency. It is a time consuming process, which prompted the development of a parallel algorithm to optimize and distribute the saliency detection processes that are loaded into the multi-processors. Spatiotemporal saliency is then obtained by combining the temporal and spatial saliencies to represent moving targets. Finally, a discriminative online learning algorithm was applied to generate a sample model based on spatiotemporal saliency. This sample model is then incrementally updated to detect the target in appearance variation conditions. Experiments conducted on the VIVID dataset demonstrated that the proposed visual tracking method is effective and is computationally efficient compared to state-of-the-art methods.

## Introduction

Visual tracking is an active research topic in computer vision. It has been used for many applications, such as activity recognition, surveillance, robotics, and human-computer interaction [1]. It has also been used for aerial video processing, such as tracking and object recognition, and is essential for intelligent remote sensing technologies such as unmanned aerial vehicle (UAV). In contrast to fixed cameras, aerial videos is more portable and can conduct reconnaissance and surveillance [2]. However, visual tracking algorithms and systems often fail on aerial videos. The sources of this failure include appearance variations in the target image caused by relative camera and target motion and inadequate spatial resolution or noise, scale changes, and pose variations [3–5]. The explicit modelling of target appearance provides one approach to deal with the problem of the variation of the target's appearance during tracking. Usually, appearance modelling subsystems are composed of modules that provide a visual representation and a means of updating the model. [6]. The visual representation significantly influences the performance of appearance modelling due to changes in target appearance in the images. A suitable representation could use visual properties, such as color, texture, intensity gradients, and saliency to represent the targets and other objects in the scene. The represented targets can be incrementally updated based on the updated model to generate sample model of target [7]. Therefore, an efficient visual representation is crucial to describe the target in the scene and generate a sample model [4,8].

Recently, biological features reported promising results in computer vision systems. With recent development involving biological features, visual saliency detection have attracted the attention of researchers for extracting Attentional Regions (AR) in the images [9]. The visual saliency detection is inspired by biological human mechanisms, specifically eye mechanisms and vision fixation, indicating that human perception is sensitive to more salient regions [10,11]. The salient regions in the image are called saliency. Based on the visual saliency detection and AR extraction, various studies have been carried out to detect moving objects in videos. The visual saliency detection methods for moving object detection can be categorized into temporal, spatial, and integrated (spatiotemporal)-based methods. Temporal saliency is generally used to extract the motion cues in videos. However, temporal saliency detection alone is not efficiently able to detect the moving regions due to the lack of spatial information, leading to missing detail of the target appearance representation [2]. However, spatial-based saliency detection are mostly used to process static images [2]. Therefore, the temporal and spatial saliencies can be integrated and called spatiotemporal saliency, which is capable of effectively detecting moving regions.

Spatial saliency detection is the main task in spatiotemporal saliency, as it deals with the target’s visual representation. Numerous spatial saliency detection methods have been proposed in literature, based on multi-scale image features [11], graph-based visual saliency (GBVS) [12], quaternion discrete cosine transform (QDCT) [13], Fourier Transform (FT) [14], frequency-tuned [15], and integrated visual features [16]. Although various spatial saliency detection methods have been proposed, it is still necessary to improve its efficiency in dealing with target appearance variations. This improvement also needs to account for processing time, since visual tracking require quick image processing [2,6]. The current spatiotemporal saliency detection methods are only used to detect moving targets in simple scenarios, and did not account for appearance variation difficulties. This difficulty can significantly influence target detection for visual tracking performance, and neglecting this aspect could result in misidentification of targets. This paper focuses on spatiotemporal saliency detection to deal with the appearance variation difficulties in aerial videos, including a proposed spatial saliency detection method for visual target representation.

The updated model is essential for appearance modelling. It basically uses adaptive methods to deal with appearance variations [7]. The adaptive methods require online algorithms that can be learned and updated incrementally [7]. On-line learning algorithms are categorized into generative-based and discriminative-based methods. The former are mainly focused on how it can fit models from the target [6,7], with examples being Gaussian Mixture Models [17,18], kernel density estimation [19], and subspace learning [20,21]. The discriminative-based methods concentrates on binary classification, and is able to classify objects in the scene into target and non-target regions discriminatively. Some discriminative-based methods boosts [22,23] Support Vector Machine (SVM) [24], randomized leaning [25], discriminant analysis [26,27], and code book learning [28]. Discriminative online learning methods can increase the efficiency of online predictive performance results compared to its generative counterpart [6,7].

Several visual tracking methods have been proposed based on appearance modeling. This paper reviews the existing related methods tabulated in Table 1. It will also address the visual representation and update model for each method. Details of the flow for study design and search strategy through the review have been provided in S1 File and S2 File as supplementary materials.

**Table 1:** Review of some related methods.

Methods	Visual representation	Model Update	Advantages/disadvantages
Zhang et al., [3]	Mean shift color segmentation, Dense Optical flow estimation, affine transformation calculation to represent large segments, pixel-based Subordinate degree calculation for segment representation.	Multiple background model estimation, updating model by merging similar background models.	The proposed method is able to detect the moving targets in complicated conditions, moving camera and by multi-model background estimation. However, Optical flow-based visual representation are high computational cost. Low processing speed (4s per frame.).The proposed method is for fixed target size and is not able to detect targets with different size.
Xianbin et al., [57]	Kanade–Lucas–Tomasi (KLT) features for ego motion estimation, Using motion consistence, background Kanade–Lucas–Tomasi features are separated, and a target is represented. Incorporation of camera ego motion and particle filter to represent the target position.	Ego camera motion model is constructed based on background features. In order to update the model HSV color histogram and Hu moment are utilized.	The proposed method is able to track the targets in airborne videos when the camera and target are moving. However, the appearance modeling in this visual tracking method is able to detect the moving target in simple background. Since it is not included online learning model updating, it is difficult to extend the application of this method in complicated conditions such as occlusion and illumination changes.
Aeschliman et al., [58]	SURF-based feature Segmenting the target from the background.	Spatial distribution of the corresponding pairs in the images with background modeling	The proposed method is able to construct an accurate background model to target tracking when both camera and targets are moving. It is able to track the targets when appearance variations caused by shadows and lighting changes. However, prior parameters setting for camera calibration are required. Manually initialization of target representation is required in the tracking process.
Shen et al., [2]	Multi cue spatial-color sub-regional distribution. Histogram-based (color) contrast. Spatiotemporal saliency target representation.	No background or target appearance modeling.	The propose method is fast and able to detect the moving target when the camera and target are both moving. However, there is no melding of background and not efficient in complicated conditions such as cluttered-background, occlusion and illumination. High false alarm rate in appearance scenarios.
Yu et al., [59]	Optical flow, Tensor Voting	Background modeling	The proposed method is able to detect the moving targets efficiently in noisy background and long-term occlusions. However, the proposed method is not included spatial information for target representation; which is not able to describe the details of target appearance.
Lan et al., [52]	Kanade-Lucas-Tomasi (KLT) feature, Relative distance change (RDC) measure to represent the target in background scene that is based on a classification of matched feature pairs	No background or target appearance modeling.	The proposed method is fast and accurate in moving object detection in airborne Video. Relative distance change (RDC) measure is proposed to distinguish the target from background scene, which is invariant to image rotation, translation, and scaling. However, There is no melding of background and target, and it is not efficient in complicated conditions such as cluttered-background, occlusion and illumination.High false alarm rate in appearance scenarios.

This paper proposes a spatiotemporal saliency and discrminative on-line learning method for handling appearance variations in visual target tracking in aerial videos. The temporal saliency is used to extract moving target regions based on frame differences and Sauvola thresholding algorithm. The spatial saliency is used to represent the target appearance representation for the extracted moving regions. In the case of spatial saliency detection, SLIC superpixel segmentation, color, and moment features are used to compute region uniqueness and spatial distribution of saliency measurements. However, it is a time consuming process, and a parallel algorithm is proposed to deal with it. The algorithm is based on region (extracted moving regions) distribution in a multi-core platform. Spatiotemporal saliency is then obtained by combining the temporal and spatial saliencies to represent moving targets. Finally, a discriminative online learning algorithm is applied to generate sample models, which are then incrementally updated to detect the target in appearance variation conditions. The details of the proposed method will be elucidated in the materials and methods section. The contributions of this study are as follows:

1. A spatial saliency detection method is proposed to effectively represent the target appearance based on region uniqueness and spatial distribution measurements.
2. A parallel spatial saliency detection algorithm is proposed and implemented in a multi-core platform to enhance the processing-time for the spatial saliency detection process.
3. A spatiotemporal saliency and discriminative on-line learning method is proposed for visual target tracking in aerial video to overcome the difficulty of moving target detection in appearance variation conditions.

The rest of this paper is structured in the following order: materials and methods detail the proposed methods of this work. Section 4 discusses the experimental results and performance evaluation. Finally, Section 4 presents the conclusion.

## Materials and methods

**Fig 1:**
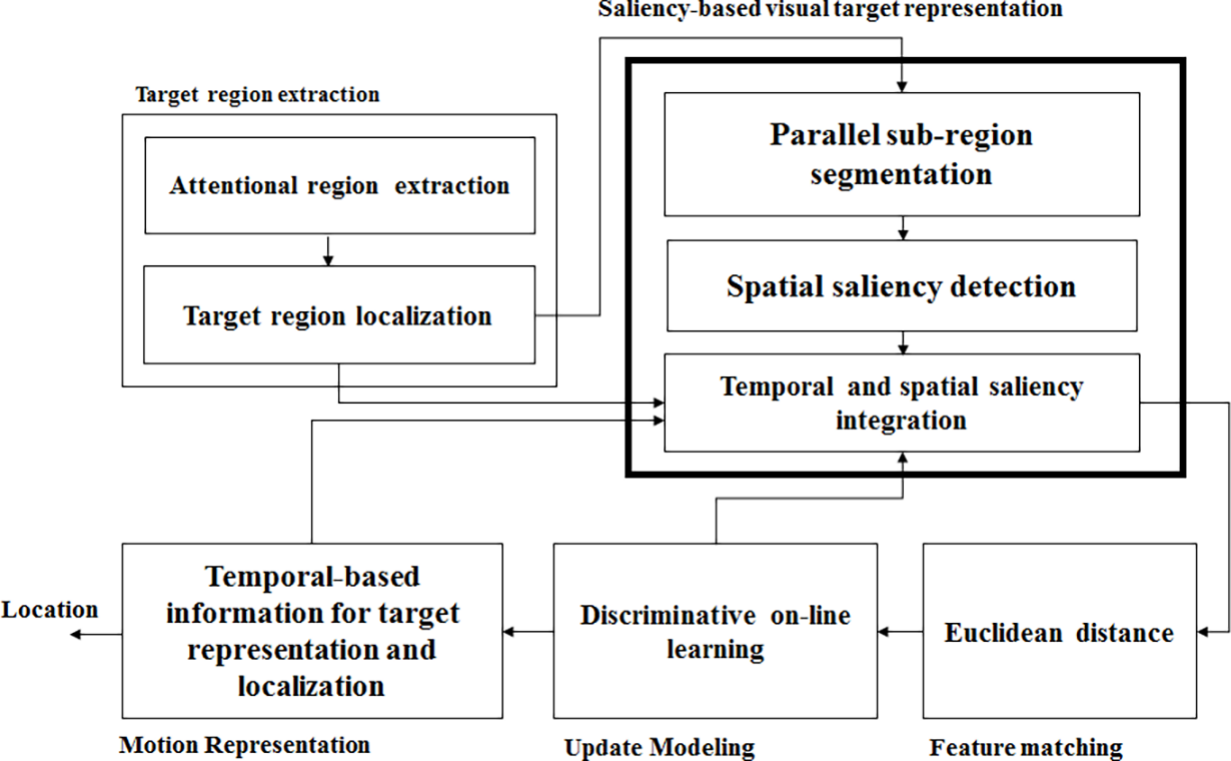
Our proposed framework.

This section discusses the proposed methods outlined in this work. It consist of modules, which are target region extraction, saliency-based visual target representation, feature matching, target motion representation, and update modelling, as per Fig 1. This research work has been conducted and reported according to PRISMA checklist guideline (refer to S3 File) to follow the best practices in systematic review reporting. The details of the proposed method are detailed in the following subsections.

### Target Region Extraction (TRE)

The TRE module involves two sub-modules: temporal saliency detection and target region localization.

#### Temporal saliency detection

Moving regions attracts more attention in videos [10]. These regions are generally called AR [29]. AR is extracted using temporal saliency information, upon which they are called candidate mask (CM) regions. To obtain CM regions, frame difference and Sauvola local adaptive thresholding algorithms are used alongside the following details:

***Frame Difference***. The frame differencing algorithm is used to identify moving objects in consecutive frames. This basic technique employs the image subtraction operator, which takes two images (or frames) as input to produce an output [30]. Eq 1 can be used to calculate the difference between the two frames:

$$\left(DIFF[i,j]=I_{1}[i,j]–I_{2}[i,j])\right)$$ (1)

where I_1_ and I_2_ are two subsequent image frames, and *i* and *j* are pixel coordinates for each frame.

**Fig 2:**
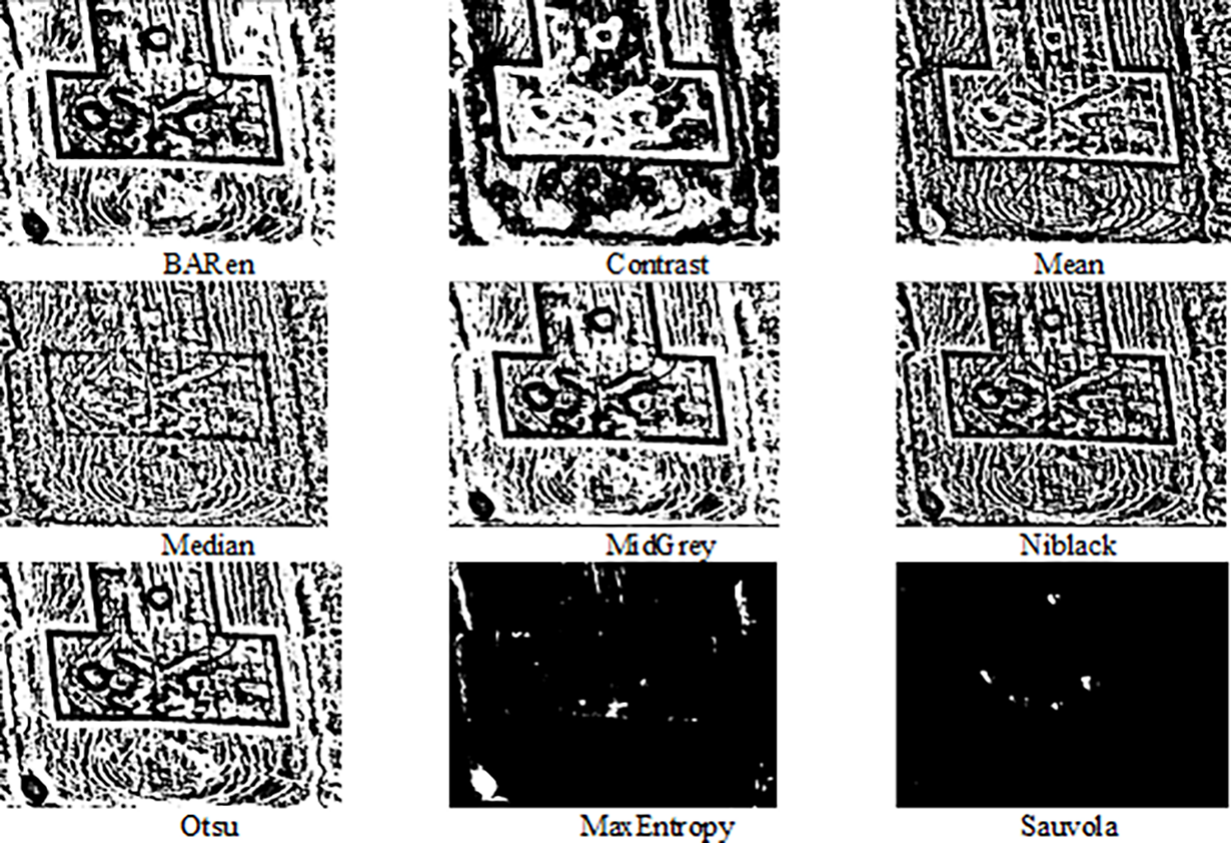
Visual comparison for thresholding algorithms.

***Image thresholding*,** The result of frame difference included noises as well. A local adaptive thresholding, in the form of Sauvola algorithm [31], was utilized to threshold the image and remove unwanted regions (noises). In order to show why we used the Sauvola thresholding algorithm, an experiment was carried out, and the comparison results presented. As illustrated in Fig 2, the result of Sauvola thresholding algorithm is better than other algorithms in the context of the number of noises. The Sauvola algorithm was shown to satisfy performance in noisy images, as per [32,33]. Based on the obtained results from the experimental and previous studies, the Sauvola local adaptive thresholding algorithm is used for thresholding and segmentation purposes.

#### Target region localization

Once the temporal saliency module has identified the CM regions, the localization module is applied to localize the extracted CM regions based on connected component and blob identification algorithms [34]. This module involves the following steps:

***Edge segmentation*.** Canny edge segmentation is ran on the binarized image to further improve the extracted region [35].

***Blob Identification***. The output of the edge segmentation contains many pixels and regions. Most of them are unwanted and needs to be removed. A blob analysis can be used to remove them, and is performed based on the connected components and region properties.

**Fig 3:**
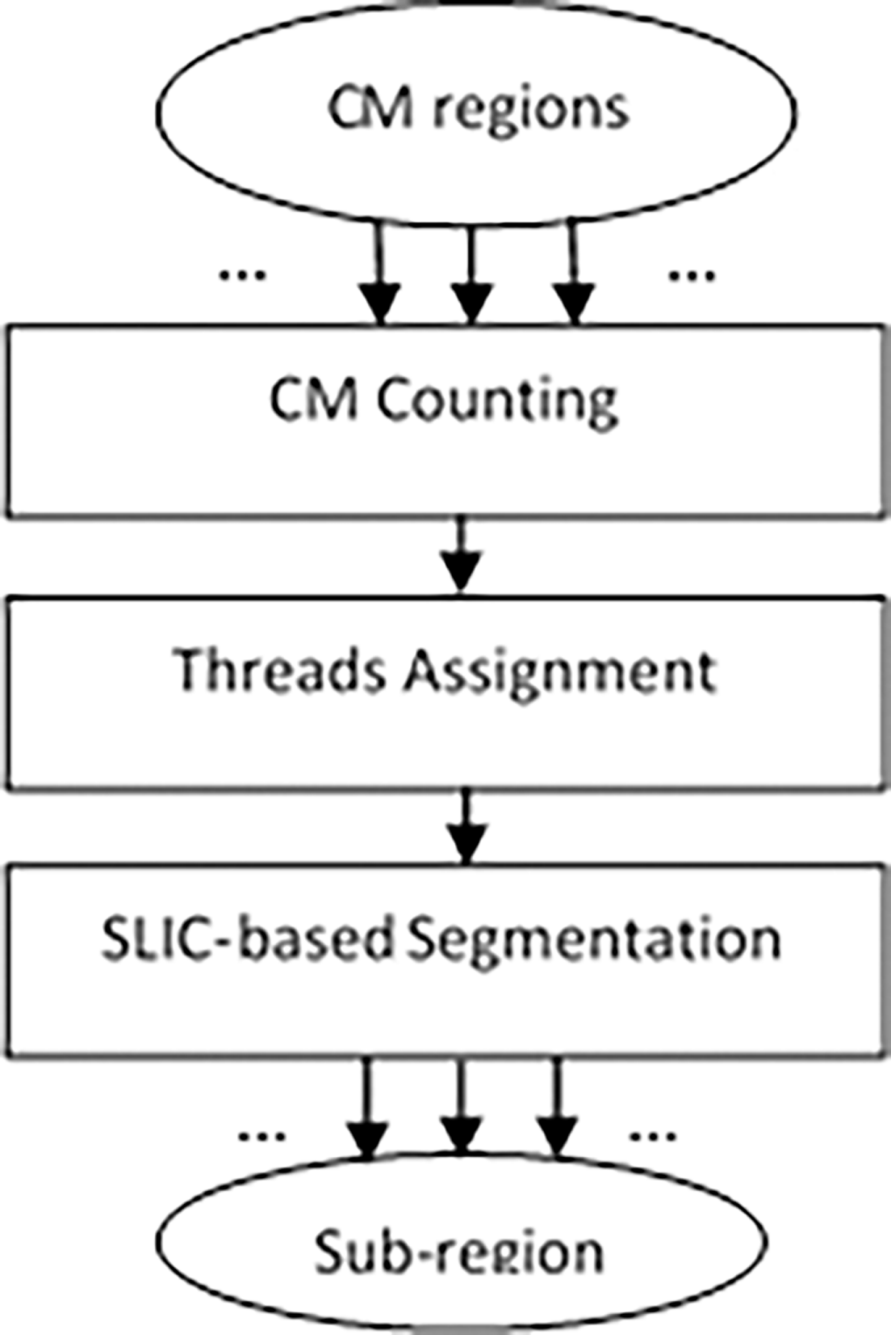
Candidates mask generation.

***Candidate Mask (CM) Generation***. The area and centroid features are used to recognize the location of each ROI. The ROI uses X_pos_ and Y_pos_ as the centroid coordination of each region that can be obtained using moment features. Based on the obtained value of the centroid coordination and blob region size identification, the candidate mask (CM) are generated, as shown in Fig 3.

### Saliency-based Visual Target Representation (SVTR)

SVTR represent the target appearance. It can be used for target detection and target sample model generation. Saliency-base features have been investigated by many researchers for target detection due to its high performance [2]. By adopting the visual saliency detection issue, this paper proposed a visual saliency detection to represent targets in aerial videos. SVTR consist of two steps: sub-region generation and spatial saliency detection, detailed in the following subsections.

#### Parallel candidate mask segmentation

**Fig 4:**
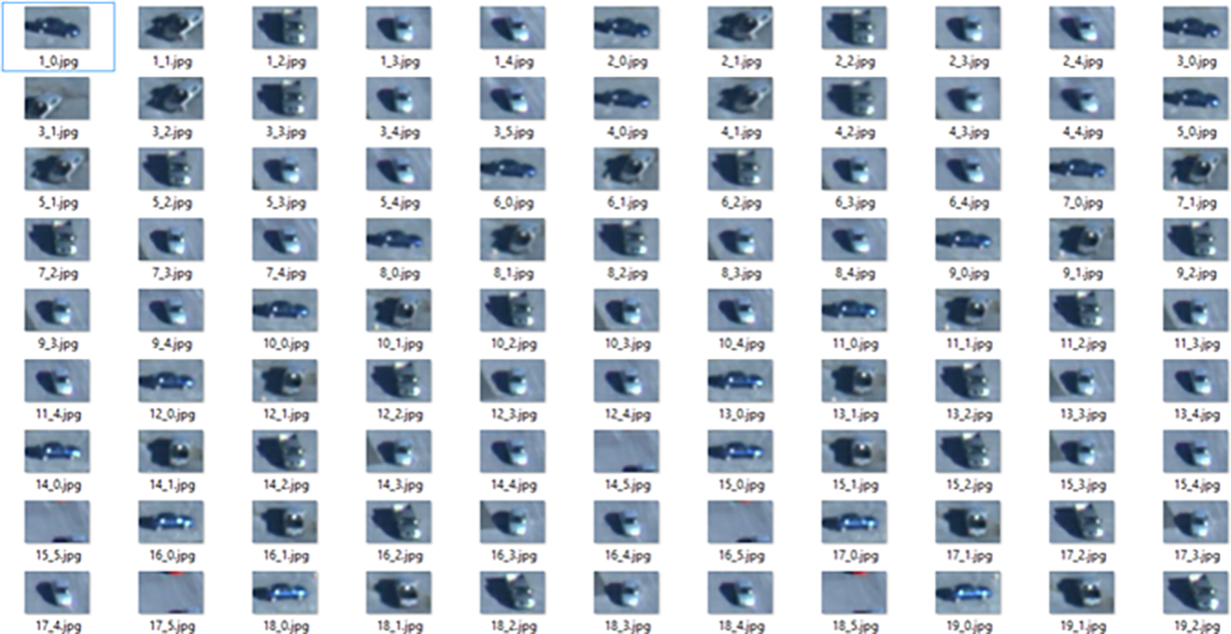
Parallel algorithm for candidate mask segmentation.

The purpose of this step is to segment the CM region into sub-regions and distribute it into different processors. The sub-regions are used to distinguish the non-target region and target region, examples being the 4_5.jpg and 16_5.jpg images shown in Fig 3. These images are non-target regions that can be distinguished using sub-regions segmentation. The SLIC algorithm [36] can be used for sub-region segmentation, but it is computationally expensive [37] and is time consuming in the case of spatial saliency detection. To circumvent these drawbacks, an algorithm is proposed for the distribution of the CM regions into different CPU platforms so that they can be processed in parallel. The core concept of this algorithm is to perform the segmentation process on the candidate mask region in parallel instead of the whole image. In this work, the SLIC algorithm is applied to the CM regions instead of the whole image in our algorithm. The proposed algorithm can very well decrease the computation cost of the SLIC algorithm. Fig 4 shows the proposed algorithm for parallel candidate mask segmentation.

1. *CM regions Counting*, this step counts the number of CM regions.
2. *Threads Assignment*, the number of threads are identified based on number of CM regions. Then, each CM region is assigned to a thread.
3. *SLIC-based Segmentation*, SLIC is used to segment the CM regions and generate sub-regions.

**Fig 5:**
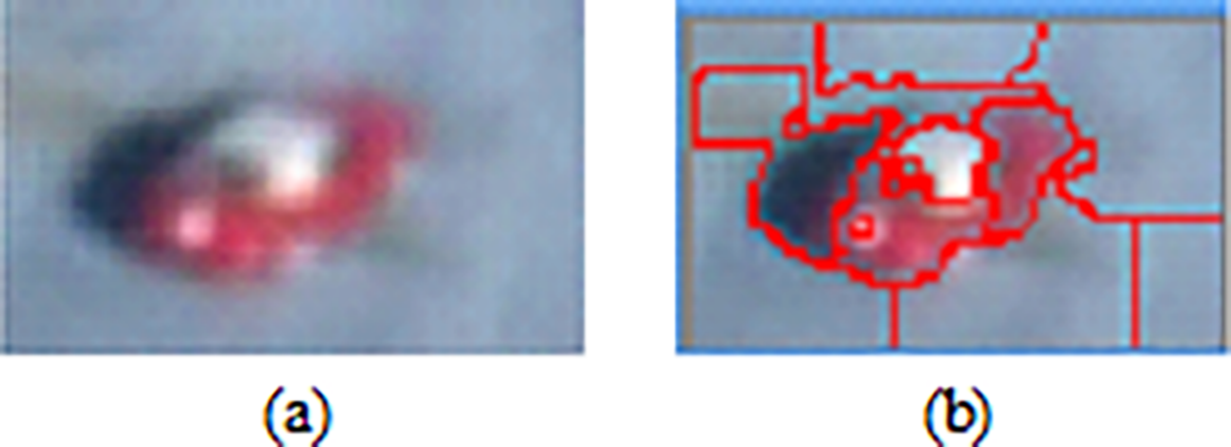
Segmented sub-regions using SLIC. (a) A candidate mask (CM) region, (b) Sub-region generation based on proposed parallel SLIC segmentation algorithm.

In SLIC-based segmentation, the proposed algorithm can be extended to the usage of the SLIC algorithm in video-based on parallel implementation [38]. Based on the SLIC and parallel algorithm, the CM regions can be segmented to generate sub-regions, as per Fig 5.

#### Spatial saliency detection

This section details the detection of more suitable sub-regions based on spatial saliency. In order to detect spatial saliencies, region uniqueness and spatial distribution (compactness of regions) measurements are investigated. Color and moment features are used for uniqueness and compactness measurements. The color contrast feature is used for dissimilarity measurement of a sub-region compared with its neighbor regions, and the moment feature is utilized to measure the compactness of two different sub-regions (distance distribution between sub-regions). Details of uniqueness and compactness measurement are as follows:

*1) Spatial uniqueness measurement*. The uniqueness for a sub-region was measured to determine if it exhibit similar color contrast with its neighbors’. The color feature for both regions are then extracted, and their similarities are measured using an Earth mover’s distance (EMD) algorithm. Eq 2 utilizes the EMD to measure color similarity measurement in the following form:

$$C_{i}=\sum_{j=1,j\neq i}^{n}exp(\frac{{-D}_{i,j}}{a_{j}})$$ (2)

where a_j_ is the area of region R_j_, and D_i, j_ denotes the EMD to measure the distance of the mean color between R_i_ and R_j_. Eq 2 indicates the regions whose colors are different from other regions in the image. The color similarity measures of all regions are then normalized into the range of [0, 1], and the color saliency of R_i_ is interpreted by $S_{i}^{col}=1-C_{i}$. Higher color saliency values are assigned to regions where higher color dissimilarity is recognized compared to other sub-regions.

*2) Spatial compactness measurement*. The pixels of the those sub-region that included high saliency values are used to determine compactness [39]. Compactness is defined when two individual sub-regions are close to one other. Spatial moments feature is used to measure the compactness of the sub-regions. First and second-order of moment feature is used for spatial moment feature [40]. The Raw moment *FD*(*m*, *n*) is used to calculate the moment features. Accounting for the fact that $\bar{m}$ and $\bar{n}$ are components for the region centroids, the *FD*(*m*, *n*) for (*p* + *q*) can be defined as [3,16],

$$M_{pq}=\sum_{p}\sum_{q}m^{p}\;n^{q}\;FD\;(m,n)$$ (3)

Considering the *FD*(*m*, *n*) as 2D continuous function, the moment feature can be calculated using Eq (4),

$$M_{pq}=\iint m^{p}\;n^{q}\;FD(m,n)$$ (4)

where the centroid coordinates can be calculated as:

$$\bar{m}=\frac{M_{10}}{M_{00}}\;,\;\bar{n}=\frac{M_{01}}{M_{00}}$$

Then, the obtained values (region centroids) from the moment feature are used for sub-region compactness measurement. The sub-region compactness is measured based on distance measurement of identified center points of two spatial moments. Eq 5 can be used to measure compactness.

$$D_{i}^{s}=\sum_{j=1}^{N}{\left\|p_{i}-p_{j}\right\|}^{2}$$ (5)

where the ‖p_i_ − p_j_‖^2^ is a quadratic term of distance between the centroid of sub-region i and j.

#### Saliency integration

The meaningful integration of temporal and spatial saliencies is necessary to produce a final spatiotemporal saliency map [41]. In this paper, adopted from [16], the final saliency map is generated by integrating the temporal and spatial saliencies.

### Feature matching

During the spatiotemporal saliency detection, the generated features based on color and moments features are integrated, and a feature vector is generated for individual sub-regions. The generated feature vector for new sub-regions are compared with previous generated feature vector extracted from prior frames. An Euclidean distance is used to measure the difference between these two feature vectors [42]. Based on the obtained value from the Euclidean distance, it can be surmised whether or not a new sub-region belongs to a target region. The recognized sub-regions are used as targets for target motion representation and model update.

### Target motion representation

This module localizes the target and represent the motion features of the moving target region. A tracking and detection algorithm, adopted from [43], is used for target motion representation [40]. The tracking and detection algorithm is based on the output from the spatiotemporal saliency and a blob region extraction algorithm. The output of the spatiotemporal saliency, which consist of temporal saliency extraction, is integrated with blob analysis to localize the targets in the videos.

### Model update

The recognized target region derived from the feature matching process are used to generate a model to represent the targets in appearance variation conditions. This model also requires incremental update to obtain more samples from target appearance changes.

This study adopts a Multiple Instance Learning (MIL)-based algorithm to generate and update the model. Principally, the MIL algorithm requires instances (image patches) and discriminative classifier to classify and label the instances into positive (target regions) and negative (non-target region) [44,45]. The former is then collected into a set called bags, which are incrementally updated through on-line discriminative classifier to distinguish the positive and negative instances.

**Fig 6:**
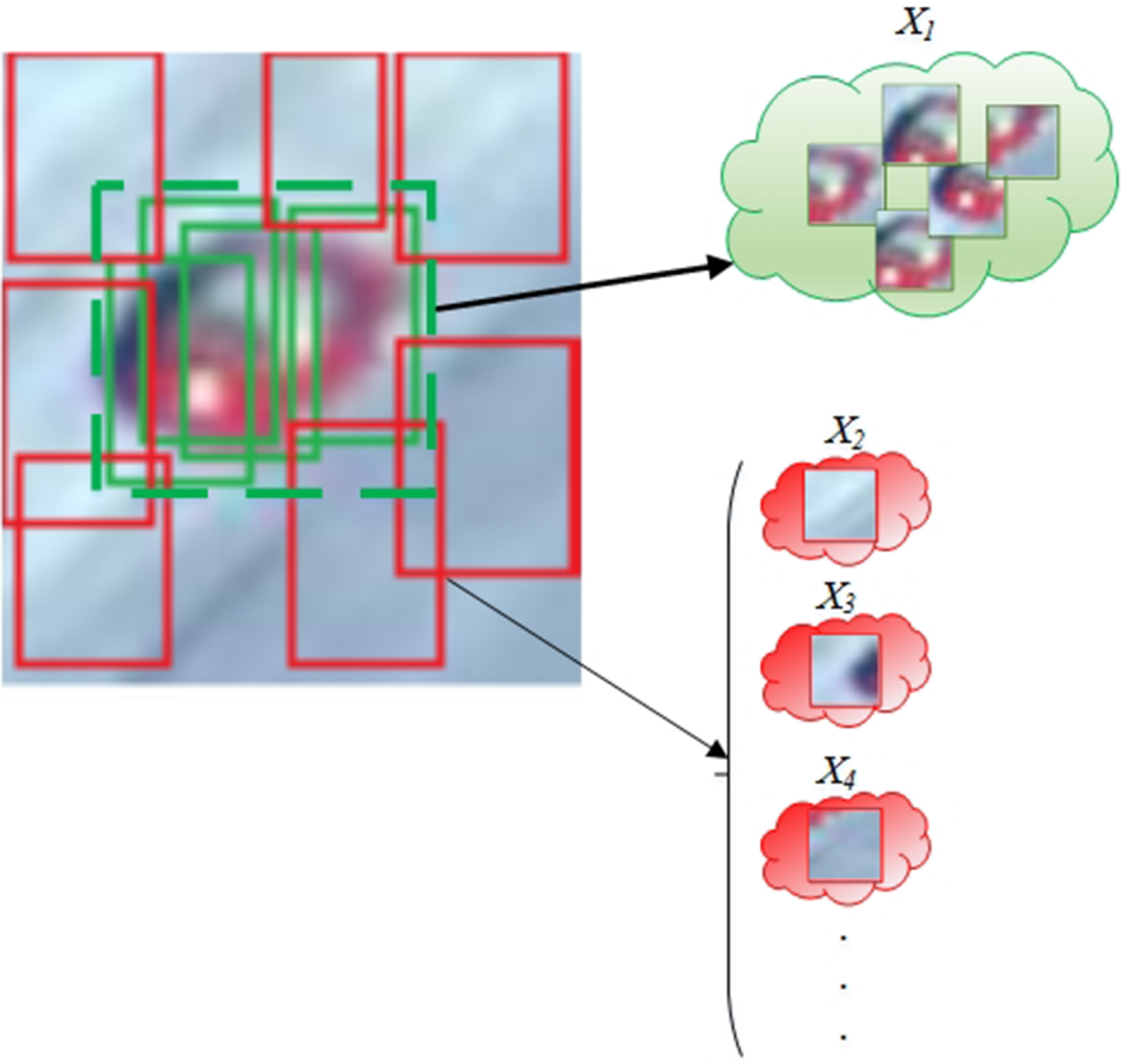
Labelling of positive instances in a bag and negative ones for a particular target.

In this study, the positive instances are defined as different parts or a whole region of target, while the negative instances consider the regions surrounded around target region belonging to the background. In order to distinguish the positive and negative instances, the extracted features from the feature matching process are used for each instances. The features from new instances are compared to existing features from previous instances, which were already located in the bag. A similarity measurement based on template matching algorithm was carried out for this comparison. This matching process is used for instances classification to identify and label the positive and negatives instances. Finally, the positive instances are collected into bags. These bags could contain many samples from the target regions, which can be incrementally updated for more samples. Fig 6 shows an example of generated bag (positive instances) and negative ones for a particular target. The positive instances are collected in the bag and labeled X_1_, while the negative instances are labeled X_n._ For appearance variation such as pose and scale, the update modelling is also performed to generate the models. The generated model is updated to cover the pose and scale variations of target in upcoming frames.

## Experimental results

This section details the experimental results and performance evaluation of the proposed method. The proposed method was used various standard videos to confirm its efficiency. The videos are collected from VIVID dataset [46], and report appearance variation difficulties, such as complicated background, illumination changes, scale changes, and pose variations. The results from the videos are visually and quantitatively compared to those outputted by other methods. The visual comparison reports the image results by the proposed and other methods, while quantitative analysis involves performance measurements based on precision, recall, F-measure evaluation metrics, and processing time.

### VIVID dataset

The VIVID dataset consist of different types of aerial videos for visual tracking evaluation [46]. The videos are captured using a single camera mounted on an aerial device at 30 frames per second (fps). The VIVID dataset is constructed for the purpose of visual target tracking and testing, and provides a range of complicated scenarios such as arbitrary and abrupt camera motion, varying illumination, occlusions, fast-moving targets, which makes a suitable dataset for testing visual object tracking [47]. The details of the videos are shown in Table 2. These videos confirmed that the VIVID dataset is excellent for testing visual tracking method in appearance variations and complicated conditions [48] (see S1 File for more details).

**Table 2:** Details of VIVID data set.

Video	Number of frames	Image size
EgTest01	1821	640 * 480
EgTest02	1301	640 * 480
EgTest03	2571	640 * 480
EgTest04	1833	640 * 480
EgTest05	1764	640 * 480

### Visual comparison

**Fig 7:**
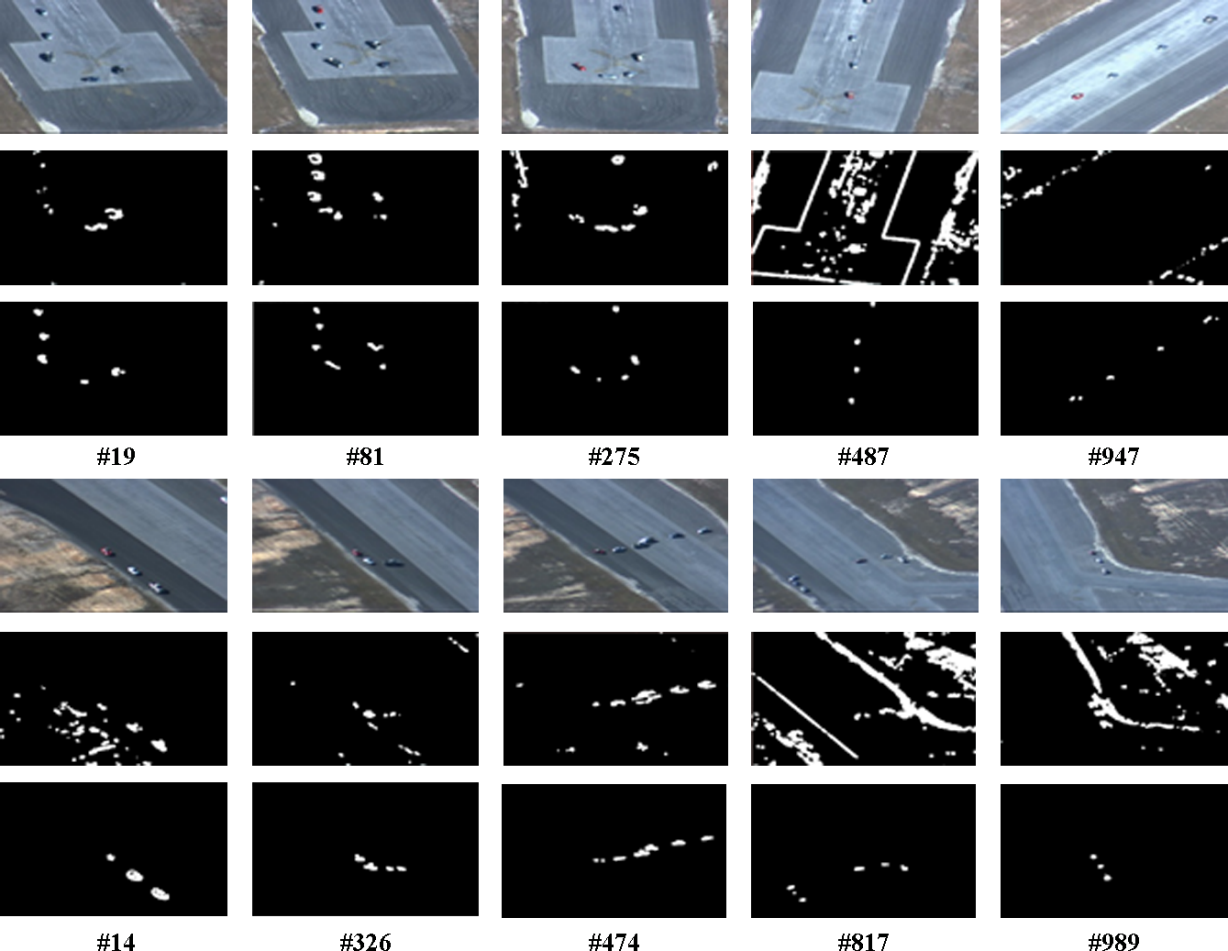
The moving target segmentation for aerial images, first row is original image, second row is the frame difference technique, and the third row is our proposed segmentation method.

**Fig 8:**
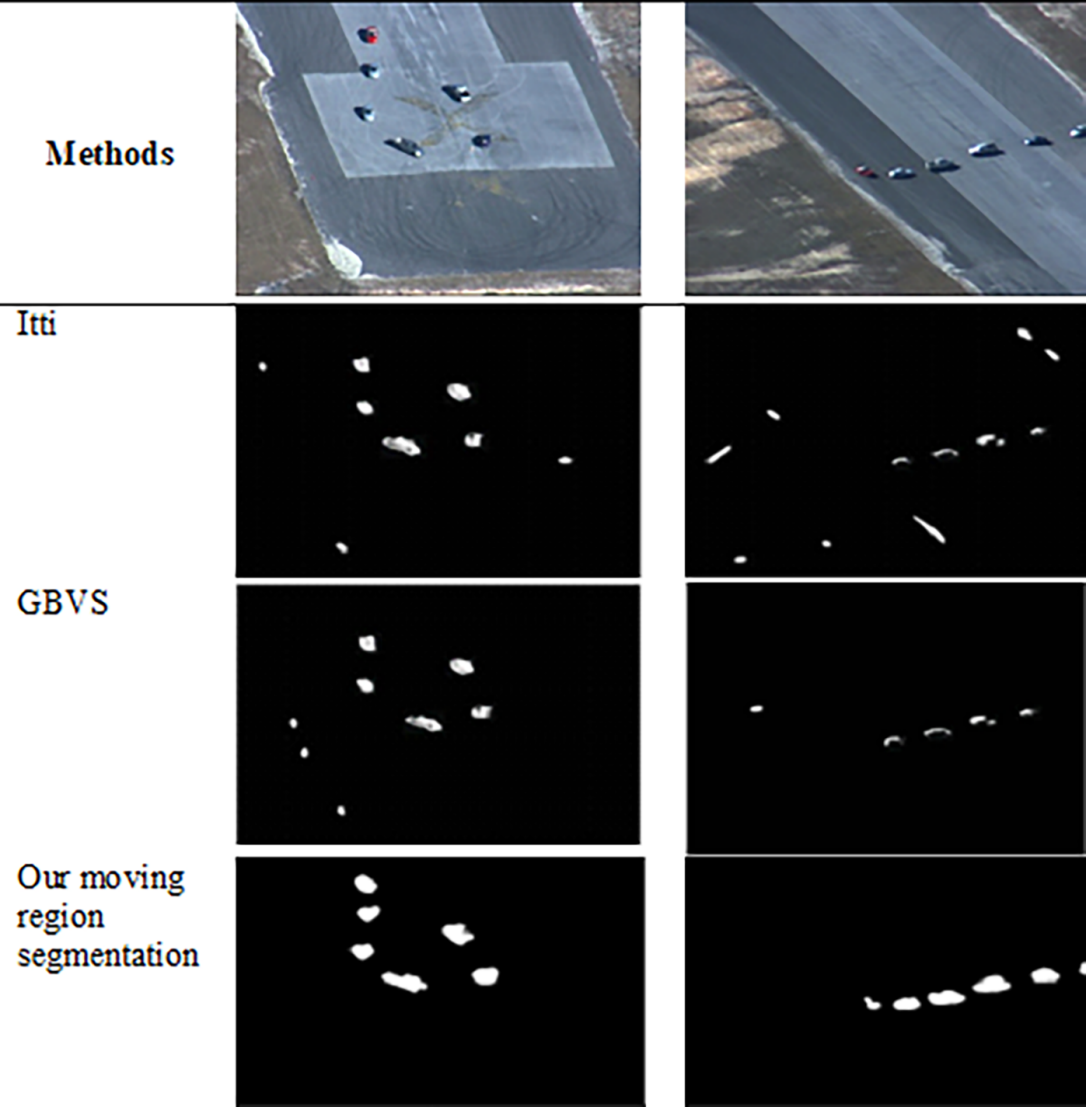
Visual comparison for moving target region segmentation for saliency-based methods and our proposed.

**Fig 9:**
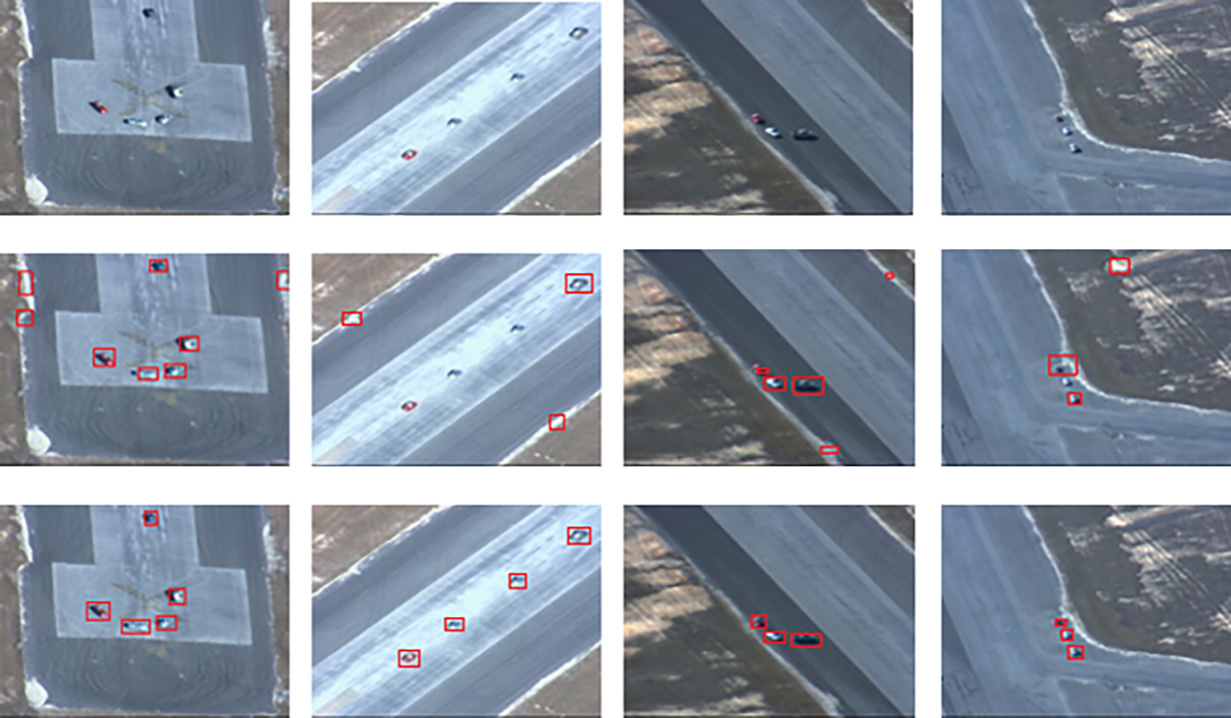
Visual comparison for moving target detection methods. The first row is original images, the second row is frame difference method and third row is our proposed method.

In this section, the visual comparison presents the result of moving target region segmentation process for the proposed method and other methods. Visual comparison was performed in two categories: region segmentation and motion-based detection. For the former, comparison were made between the proposed method and other methods: Itti [11] and GBVS [12] methods, as shown in Figs 7 and 8. The Itti and GBVS methods are considered as laying the foundation for saliency-based detection systems. They are mainly used as benchmarks for new visual saliency detection algorithms. On the other hand, the proposed method is compared to a common motion-based detection algorithm in the form of frame differencing method, as shown in Fig 9.

### Quantitative analysis

The quantitative analysis consists of performance evaluation and comparing the proposed method to other methods. In this paper, recall, precision, and f1-measure evaluation metrics are measured to evaluate its performance [48]. Basically, some variables need to be defined to measure the performance metrics, which are True Positive (TP), True Negatives (TN), False Positive (FP), False Negative (FN).

- TP: Detected salient regions corresponding to a target,
- TN: No detection of salient regions where there is not a target,
- FP: Detected salient regions that do not correspond to a target,
- FN: No detection of salient regions where there is, in fact, a target.

According to variable definitions, the performance metrics are measured using Eqs (6), (7), and (8).

i Precision,
  $$Precision=\left(\sum_{i=1}^{n}\frac{TP}{TP+FP}\right)\times 100\%$$ (6)
ii Recall,
  $$Recall=\left(\sum_{i=1}^{n}\frac{TP}{TP+FN}\right)\times 100\%$$ (7)
iii F1-measure

F-measure is regarded as an integrated performance criterion of precision and recall,

$$F_{\beta }=(1+\beta^{2})\times \left(\frac{precision\times recall}{(\beta^{2}\times precision)+recall}\right)$$ (8)

Here, we set β = 1 to calculate the harmonic mean of recall and precision,

$$F_{1}-measure=2\times \left(\frac{precision\times recall}{precision+recall}\right)$$ (9)

where the F_1_-measure is the harmonic mean of precision and recall, and is extensively used in pattern recognition community to evaluate the performance [48]. Table 3 shows the precision, recall, and F_1_-measure metrics results for the proposed method.

**Table 3:** Proposed method evaluation based on precision- recall and F_1_-measure metrics.

Video	Precision	Recall	F_1_-measure
EgTest01	96.73	98.85	97.78
EgTest02	66.00	84.97	74.29
EgTest03	80.68	84.94	82.76
EgTest04	83.91	89.32	86.53
EgTest05	68.11	82.62	74.67

The proposed visual tracking is compared with other visual tracking methods, such as the Variance Ratio [28], Color-based Probabilistic [49], and Wang et al., [50], Shen et al., [2], Yin et al., [51], Lan et al., [52], annealed mean shift (AMS) [53], Landau Monte Carlo (WLMC) [54], N-Fold Wang-Landau (NFWL) [54], and cascade mean shift (CMS) [55]. The comparisons were conducted based on the F1-measure. Table 4 shows the5quantitative comparison results for the proposed method and other relevant methods.

**Table 4:** Quantitative comparison of visual tracking methods and our proposed method based on F_1_-measure.

Video	Variance Ratio	Color-based probabilistic	Wang et al.	Liang et al.	Shen et al.	Yin et al.,	Lan et al.,	AMS	WLMC	NFWL	CMS	The proposed method
EgTest01	68.32	65.03	72.53	76.78	96.30	93.06	91.87	84.12	68.47	63.85	84.72	**97.78**
EgTest02	56.67	65.24	53.30	60.81	73.31	61.40	47.63	76.18	62.82	59.85	78.14	**74.29**
EgTest03	77.16	65.08	85.84	77.39	71.12	60.68	48.39	71.78	61.14	58.51	72.63	82.76
EgTest04	84.61	59.71	83.52	81.40	65.31	52.29	70.08	68.62	53.04	52.73	74.54	**86.53**
EgTest05	82.01	71.15	83.87	80.56	50.13	75.62	71.96	63.96	58.80	56.75	70.08	74.67

**Fig 10:**
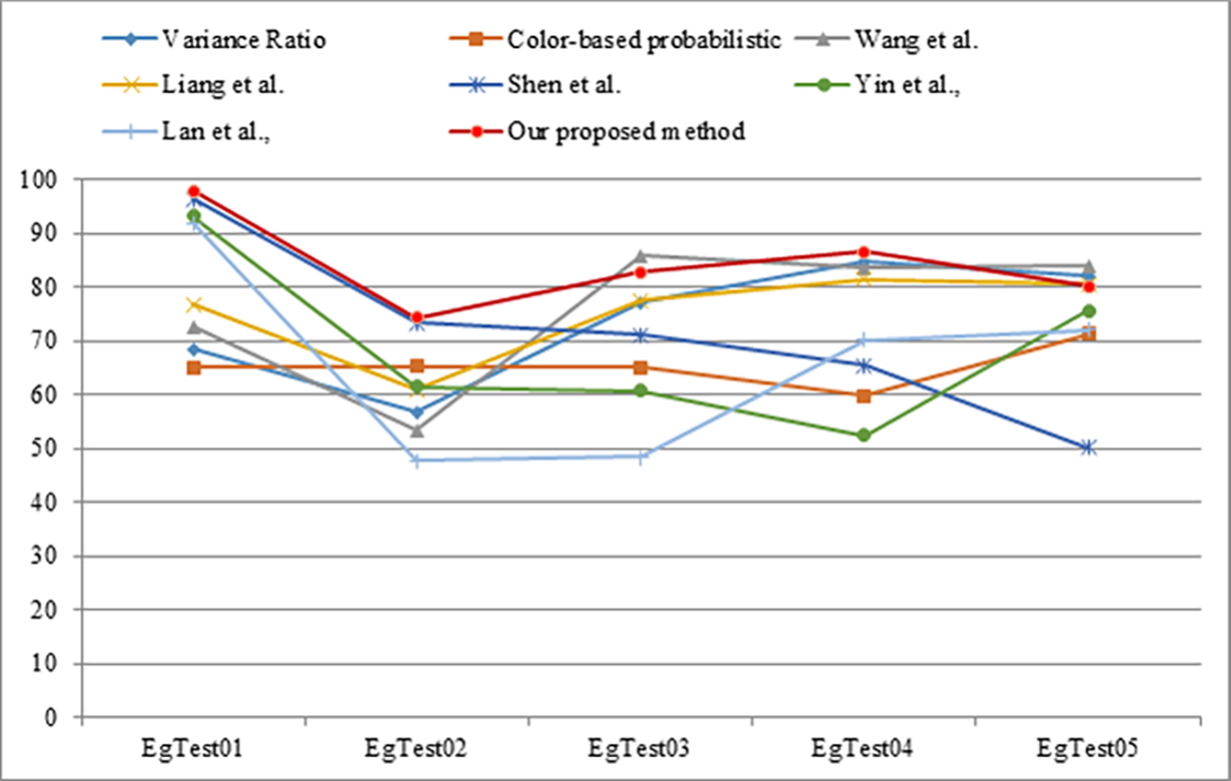
Illustration of quantitative comparison for visual tracking methods and ours.

On average, the proposed method achieves comparable performance compared to other visual tracking methods within this dataset. In some sequences, our method outperformed other techniques; in sequences 3 and 5, the presence of occlusion and overlap led to Wang's method performing slightly better as shown in Fig 10.

Furthermore, Youden’s test is also accomplished on achieved results to prove the efficacy of the proposed method. The Youden’s test was introduced by Youden in 1950 [56] which is a measurement to statistically analysis the performance of the algorithms and methods. Principally, this measurement is utilized *J* variable for performance analysis. This *J* variable can be calculated using following equation:

$$J=\frac{TP}{TP+FN}+\frac{TN}{TN+FP}-1$$ (10)

**Fig 11:**
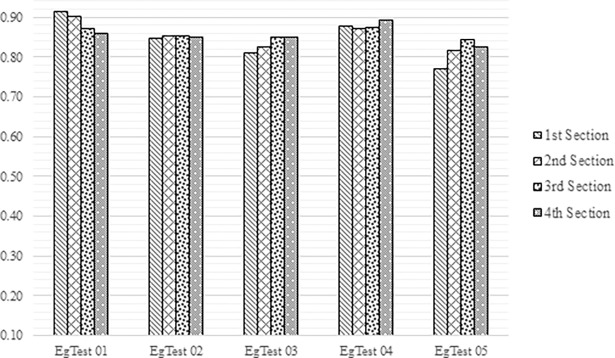
Youden’s J values for each EgTest videos and their sections.

In this experiment, the Youden’s *(J)* measurement were calculated for each video (EgTest(s) frame sequences) separately. For this measurement, each video was firstly divided into four sections to test the performance in different range (number of frames). For example, EgTest02 contains 1821 frames in total. It was divided to four sections as 150, 450, 1150 and 1821 number of frames. For each section, the Youden’s *J* value was calculated for each sections and videos as shown in Fig 11.

The EgTest videos have different environment complexities. These complexities include vehicles overlapping, natural objects (trees) occlusion and very small vehicles. The complexities can be directly influenced on detection results. With increasing the complexities, they lead to increase false negative and decrease true negative. For example, if the targeted vehicle tries to pass front another vehicle, an overlapping complexity can be occurred. In this case, the overlapped vehicles as salient region and target cannot be detected and then it is caused to increase false negative. Considering the complexity issue, Youden measurement shows that our method has better results on EgTest01 and EgTest04. These two videos have less complexity in compared to other videos. EgTest05 obtainedn less Youden’s J value that relates to its environment complexity. Finally, the experiments for the methods were conducted on a platform with an Intel Core 2 Quad Core 2.83 GHz CPU with 4 GB of RAM. The processing time was measured based on wall-clock time computation [38]. The tick_count class from a wall-clock (located in tbb/tick_count.h) is used to measure the wall-clock. The average processing time for our proposed method is 38.61 ms.

## Conclusion

A spatiotemporal saliency and discriminative on-line learning method was proposed for handling complicated conditions and appearance varitions in visual target tracking for aerial video. We used visual saliency-based detection to represent visual targets. Temporal saliency was used to represent the moving target regions, and is extracted based on frame difference with Sauvola local adaptive thresholding algorithms, while spatial saliency was used to represent the target appearance details in candidate moving regions. For the spatial saliency detection, SLIC superpixel segmentation, color, and moment features were used to compute the feature’s uniqueness and spatial distribution of saliency measurements. The spatial saliency detection is a time consuming process, and a parallel algorithm was derived and loaded into the multi-processors to optimize and distribute the saliency detection processes. Spatiotemporal saliency was then obtained by combining the temporal and spatial saliencies to represent moving targets. Finally, a discriminative online learning algorithm was applied to generate a sample model based on spatiotemporal saliency. This sample model was incrementally updated to detect the target in appearance variation conditions. Extensive experiments were conducted on the VIVID dataset, including 5 videos with appearance variations difficulties. The performance of proposed visual tracking was evaluated, and the results compared with the other methods. The experimental results confirmed that the proposed method is superior to the other methods. Future works can address other difficulties and challenges in visual tracking, such as the presence of more complicated backgrounds, overlapping, and out-of-plane difficulties.

## Supporting information

S1 File PRISMA flow diagram. (DOCX)

S2 File Search databases. (DOCX)

S3 File PRISMA checklist. (DOCX)
