# Correction: A parallel spatiotemporal saliency and discriminative online learning method for visual target tracking in aerial videos

**DOI:** 10.1371/journal.pone.0195418

**Published:** 2018-03-29

**Authors:** Amirhossein Aghamohammadi, Mei Choo Ang, Elankovan A. Sundararajan, Kok Weng Ng, Marzieh Mogharrebi, Seyed Yashar Banihashem

The fourth author’s name is incorrect. The correct name is: Kok Weng Ng.
