# Supplementary material for: A parallel spatiotemporal saliency and discriminative online learning method for visual target tracking in aerial videos
Source: PLoS One. 2018 Feb 13;13(2):e0192246. doi: 10.1371/journal.pone.0192246 (PMC5811006; doi:10.1371/journal.pone.0192246)
Supplement: S1 File — (DOCX) [file pone.0192246.s001.docx]

Full-text articles excluded (n = 31)

Aerial videos not used (n = 6),

Relevant results not reported (n = 9),

Appropriate metrics not used (n = 3),

Review articles (n = 5),

Cumulative follow up, or more details (n = 8).

Full-text articles assessed for eligibility (n = 68)

Records identified through database searching
(n = 228)

Additional records identified through other sources (n = 3)

Records after duplicates removed
(n = 217)

Records screened
(n = 217)

Records excluded
(n = 149)

Studies included in qualitative
(n = 37)

Studies included in quantitative synthesis (meta-analysis)
(n = 21)
