# Supplementary material for: A parallel spatiotemporal saliency and discriminative online learning method for visual target tracking in aerial videos
Source: PLoS One. 2018 Feb 13;13(2):e0192246. doi: 10.1371/journal.pone.0192246 (PMC5811006; doi:10.1371/journal.pone.0192246)
Supplement: S2 File — (DOCX) [file pone.0192246.s002.docx]

| **Database** | **Search Terms** | **Link** |
| --- | --- | --- |
| Web of Science | “Visual tracking”, “saliency detection”, “temporal saliency detection”, “spatial saliency detection”, “spatiotemporal saliency detection”, “aerial visual tracking”, “Appearance modelling”, “parallel processing”. | http://www.webofknowledge.com/ |
| IEEE | “ Aerial visual tracking”, “saliency detection”, “spatiotemporal saliency detection”, Online discriminative-based visual tracking”, “temporal saliency detection”, “spatial saliency detection”, “real-time moving object detection”, “appearance modelling”, “parallel processing”. | http://ieeexplore.ieee.org/Xplore/home.jsp |
| Springer | “Visual tracking”, “saliency detection”, “temporal saliency detection”, “spatial saliency detection”, “spatiotemporal saliency detection”, “aerial visual tracking”, “Appearance modelling”, “parallel processing”. | https://link.springer.com/ |
| Scopus | “ Aerial visual tracking”, “saliency detection”, “spatiotemporal saliency detection”, “real-time moving object detection”, “appearance modelling”, “parallel processing”. | https://www.elsevier.com/solutions/scopus |
| MDPI | “Visual tracking”, “vision-based saliency detection”, “spatiotemporal saliency detection”, “aerial visual tracking”, “appearance modelling”. | http://www.mdpi.com/ |
